# Effectiveness analysis and value incommensurability

**DOI:** 10.1186/s12962-025-00624-w

**Published:** 2025-04-23

**Authors:** Anders Herlitz

**Affiliations:** 1 Department of Philosophy, Lund University, Lund, Sweden; 2 Institute for Futures Studies, Stockholm, Sweden

**Keywords:** Value incommensurability, Effectiveness analysis, Multi-dimensional value representations, Discrimination

## Abstract

This paper argues that in many contexts where effectiveness analysis such as benefit-cost analysis and cost-effectiveness analysis is used, we have good reason to think that some benefits or costs are incommensurable in value such that neither can be determined to be better than the other, although they cannot be determined to be equally good either. Two responses to such value incommensurability are outlined: abandoning conventional ways of measuring benefits and costs and replacing one-dimensional measures with multi-dimensional measures or sticking to conventional ways of measuring benefits and costs and accepting that whatever valuation one comes up with, it will fail to reflect the actual values and value relations between benefits and costs. Both responses are argued to be problematic.

## Introduction

This paper looks at what it would mean for effectiveness analysis such as Benefit-Cost Analysis (BCA) and Cost-Effectiveness Analysis (CEA) to accept that some benefits and/or costs might stand in non-conventional comparative relations to each other, i.e., that some benefits and/or costs are “incommensurable in value” (I will return to, and explain in greater detail, this terminology in the next section). With a special focus on healthcare policy and planning (an area in which effectiveness analysis is widespread), the paper aims to bring attention to and explore the theoretical implications of value incommensurability in the domain of benefits and costs for systematic effectiveness analyses of policies and actions that study the relations between costs and benefits by comparing costs and benefits such as these can be expressed with one-dimensional measurements like $ equivalents and Quality-Adjusted Life Year (QALY).

## Value incommensurability

Sometimes, items that are valuable are difficult to compare in conventional ways. Neither of two items seems better than the other, but they do not seem equally good either. It can be hard to compare strawberry ice-cream and lemon tart, a successful career as a lawyer and a successful career as a health economist, and the works of Mozart and the works of Michelangelo. In some of these cases, the reason the comparisons are hard is lack of information. In those cases, gathering more information can help establish how the items relate to each other. Perhaps the lemon tart is stale, perhaps choosing a career as a lawyer means losing meaningful friendships, and perhaps it will be discovered that Mozart was a fraud who plagiarized less famous composers. In other cases, it has been suggested that the comparisons are hard because there simply is no fact of the matter of which conventional comparative relation hold between them with respect to overall goodness.

In philosophical value theory it is now widely accepted that sometimes we should accept that we cannot determine that any of the standard positive value relations (i.e., better than, worse than, equally as good as) determinately holds between two items [2, 3, 5–7, 16, 19]. However, there is, unfortunately, a lot of terminological confusion in the literature on this phenomenon. I will say that when no standard relation can be determined to determinately hold between two items, the items are *incommensurable in value*, and define value incommensurability as follows:

*Value incommensurability*: *x* and *y* are incommensurable in value if and only if it cannot be determined that *x* is better than *y*, that *y* is better than *x*, or that *x* and *y* are equally good.

This definition might seem cumbersome but has the benefit of being neutral with respect to the underlying explanation of the phenomenon. Although many value theorists accept the possibility of value incommensurability as defined above, there is strong and widespread disagreement regarding *why* it occurs. Some believe it is due to incomparability. On this view, no value relation at all obtains between the items [1, 23]. Some believe it is due to vagueness or imprecision. On this view, sometimes, the goodness of options and of evaluative standards such as overall goodness are vague or imprecise, which means that it for some items will be *indeterminate* how they relate to each other with respect to the standards [5, 6]. Some believe it has to do with uncertainty [24]. And some believe that value incommensurability is due to the fact that there exist more than three positive value relations. On this view, in addition to better than, worse than, equally as good as, items can be “on a par” (cf. Chang [8, 9, 22]. Value incommensurability as defined above can occur for any of these reasons.

A standard argument for value incommensurability is the so-called “small improvement argument” [8, 12, 13, 17, 20, 21, 23]. It asks us first to hypothesize that neither of two very dissimilar items, *x* and *y*, can be determined to be better than the other, then encourages us to image that one of these two items was improved by some very small amount, turning *x* into *x+*, and finally suggests that such a small improvement of one of the items would not change the comparative relation (see, e.g., Andersson & Herlitz [16]. If the small improvement does not change the comparative relation, then the items could not have been equally good to begin with, and if they were not equally good, they must have been incommensurable in value (because we have assumed that neither is better than the other). That *x +* and *y* cannot be equally good is easily proven: If *x* and *y* were equally good, and if *x +* and *y* are equally good, it follows that *x* and *x +* are equally good; equally as good as is a transitive relation. However, *x +* is, by definition, better than *x*, which shows that holding *x +* and *y* to be equally good leads to a contradiction.

The small improvement argument can be fleshed out in different ways. Assume, for instance, that the works of Michelangelo (*x*) cannot be determined to be better than the works of Mozart (*y*), and that the works of Michelangelo cannot be determined to be better than the works of Mozart. Now imagine that we discover an additional statue produced by Michelangelo. It is not a particularly impressive statue, but it shows that Michelangelo’s total production over his lifetime was slightly greater than what we thought. We come to accept that Michelangelo’s works are slightly more impressive than we first assumed (turning *x* into *x+*). Does this necessarily mean that we should conclude that the works of Michelangelo (*x+*) is better than the works of Mozart (*y*)? If we reject that conclusion, we must accept that the works of Mozart (*y*) and the works of Michelangelo (*x*) are incommensurable in value.

More generally, the underlying structure that makes small improvement arguments possible has been described as the “mark of incommensurability”:

Two valuable [items] are incommensurable if (1) neither is better than the other, and (2) there is (or could be) another option which is better than one but not the other [23]: 325)

If two items are incommensurable in value, they relate to each other in a way that persists at least some improvements and/or worsenings [15]. Since the relation equally as good as does not persist any improvements or worsenings, when no item is better than the other, accepting that the comparative relation persists means accepting value incommensurability.

At a general level, it has been suggested that value incommensurability with respect to an evaluative scale might appear when:

1. The [items] in question meet the standards measured by the scale in very different ways.
2. There are no gross differences in the degree to which each [item] exemplifies its own way of meeting the standards.
3. Meeting the standard in one way is not categorically superior to meeting it the other way [1]: 55).

The works of Mozart and the works of Michelangelo meet the evaluative scale “artistic output” in very different ways. The former is music, the latter is primarily statues and paintings. There is no gross difference in degree to which the works meet the standards; both are exceptional. And when thinking about artistic output, it is not true that music is categorically better than sculptures and paintings, or vice versa.

## Value incommensurability and healthcare policy and planning

CBA and CEA are of course rarely used in contexts where items that are typically discussed by philosophical value theorists are relevant. No one has to my knowledge attempted to apply CBA or CEA to dessert options, the works of Mozart and Michelangelo or very different career paths. However, the standard arguments used to establish the possibility of value incommensurability apply also to contexts in which CBA and CEA are often used. An example of such a context that I will focus on is healthcare policy and planning, where effectiveness analyses are widely used to inform decisions about how to allocate scarce resources.

To see how value incommensurability is plausibly present in healthcare contexts, it is useful to start by simply acknowledging that decision- and policymaking in healthcare contexts often actualizes comparisons of items that are very different and also valuable in different ways. Health and lack of health is naturally very important in healthcare contexts, and many items that are valuable are valuable in virtue of promoting health. But health and lack of health are very broad terms. The evaluative scale “healthy”– or “lack of health”– can be met in very different ways. Pain is different from loss of life. Physical disabilities of different kinds are very different (e.g., migraine and hangnails), and they are all very different from mental disabilities, which in turn are of course very different (e.g., depression and schizophrenia). Furthermore, some valuable items in healthcare contexts come with small probabilities (e.g., screening people for early signs of cancer), while others are almost certain; e.g., curing certain bacterial infections with antibiotics. Additionally, in many instances, one choice option might provide a large benefit to few individuals, while another choice option provides many small benefits to many individuals.^1^ At a purely intuitive level, it might thus seem likely that some of the benefits healthcare planners must take into account are incommensurable in value. Just like the works of Mozart and the works of Michelangelo are arguably incommensurable in value because they are valuable in very different ways, different items in healthcare contexts are valuable in so different ways so that they are arguably incommensurable in value.

It is also easy to come up with small improvement arguments in health contexts where overall goodness depend on the amount of health benefits generated. Healthcare provides a wide range of different benefits: it saves lives, it alleviates different kinds of discomfort, and prevents different sorts of disabilities. As long as one accepts that no benefit type is lexicographically better than– categorically superior to– the other, there will for all pairs of benefit types, *a* and *b*, exist a number *x* and a number *y* such that *xa* is not better than *yb* and *yb* is not better than *xa*. Without value incommensurability, *xa* must be equally as good as *yb*. Without value incommensurability– and, again, assuming no benefit type is lexicographically better than another– this is true for all benefit types. There is a number such that curing that number of headaches is exactly as good as saving a life. There is a number such that that number of years lived with debilitating depression is exactly as good– or bad rather– as a certain number of years lived with hangnails. There is a number such that saving that number of 100-year-olds is exactly as good as saving a 5-year-old. And so on. For all pairs of benefit types, one might ask: once one has calibrated the numbers so that neither calibrated benefit type is better than the other, would a small improvement of either option change the comparative relation? If neither of *x* headaches and a life is better than the other, is it plausible to think *x* headaches plus a dollar is better than a life? If neither of *x* years lived with debilitating depression and *y* years lived with hangnails is better than the other, is *y* years minus a second lived with hangnails better than *x* years lived with debilitating depression? If neither of *x* 100-year-olds and a 5-year-old is better than the other, is *x* 100-year-olds plus a cured headache better than a 5-year-old? A negative answer to any of these questions would establish that value incommensurability exists when different health benefits are compared.

A different kind of argument for the presence of value incommensurability in the healthcare context focuses on the kind of effectiveness analysis that incorporates concerns for inequalities by adding equity weights to the analysis. Rather than merely summing up the benefits and comparing those to the cost, this kind of approach sums up equity-weighted benefits and compare those to the cost. Typically, the equity weights are construed so that benefits to worse off individuals are ascribed greater value.

Insofar as a healthcare planner wants to take equity into account in this way, and weigh benefits with equity weights, a different kind of small improvement argument can be designed to argue that value incommensurability arises when one evaluates options based on both a desire to maximize health benefits and equity. Unless inequality aversion or maximization of benefits is lexicographically more important than the other, there will exists pairs of outcomes such that one contains more health benefits and the other has a more equal distribution of health benefits and neither outcome is better than the other. Are all such pairs of outcomes precisely equally as good? Is it reasonable to accept that a tiny improvement of any alternative in all such pairs of outcomes will change the comparative relation? If not, value incommensurability appears also at this level.

## Revising conventional effectiveness analysis

Conventional effectiveness analysis relies on ascribing one value, expressed on a scale using rational numbers, to benefits and another to costs, and then looking at the relation between these values. If value incommensurability obtains between items that should be valued on either the benefit side or the cost side, a unique rational number cannot describe the value of benefits (or costs). The simple reason for that is that it is a feature of rational numbers to rank things. These numbers differ in greatness. If unique rational numbers are used to describe the value of different things, the description will provide a conventional comparison: for all pairs of items, one will be ascribed a greater number than the other, or they will be ascribed an equally great number.

In healthcare policy and planning, it is common to rely on summary measures of health to measure the benefits of different options. The most common summary measure of health is Quality-Adjusted Life-Years (QALY). QALY measures health and health benefits by summing up quality-adjusted years lived. Each state of ill-health is provided a number between 0 and 1, where 1 is the number that describes absence of ill-health, and the lower the number, the worse the health state. These numbers are then multiplied with the years lived with the health state (by a single individual or by many different people), and the sum total expresses the QALY. QALY maximization as an ideal provides clear and unambiguous answers to all questions regarding which of two distributions of health benefits is best. All health benefits in all quantities are weakly ordered by QALY; i.e., for all pairs of health benefits in all quantities it is true that one generates at least as many QALYs as the other.

Value incommensurability is in tension with QALY maximization. If two items, *x* and *y*, are incommensurable in value, describing the value of *x* and *y* in terms of QALY means, by necessity, that one provides them inaccurate descriptions. QALY will weakly order *x* and *y*, but value incommensurability means *x* and *y* cannot be weakly ordered. Thus, if one accepts that some health benefits are incommensurable in value, one has a reason not to use QALY to measure the value of health benefits with the purpose of making effectiveness analysis. The reason is straightforward: it is inaccurate to impose weak orders on items that cannot be weakly ordered (in the next section, I will discuss the possibility that this reason is not strong enough to abandon QALY maximization).

To respond to underlying value incommensurability among benefits of different type, one might try to revise (or “extend”) conventional effectiveness analysis and develop an approach that relies on a kind of valuation of benefits that ranks some but not all options [26]. One way of doing this is by describing the value of benefits with vectors as opposed to with unique numbers, reflecting the multi-dimensionality of the underlying values.

Rather than measuring benefits in one dimension, one can measure benefits in several different benefit dimensions. Consider two different medical technologies, P and Q. They both extend the life of beneficiaries and also improve the health-related quality of life of the beneficiary for 8 years. Technology P extends the life of people benefiting from it with 10 years while improving the health-related quality of life with 0.2 for 8 years, while technology Q extend the life of people benefiting from it with 8 years while improving the health-related quality of life with 0.5 for 8 years. Relying on QALY, the health benefits of the technologies can be described with rational numbers (11.6 and 12). A QALY maximizer would hold Q to be better than P. An alternative approach would describe the benefits with vectors rather than unique rational numbers:

**Table:** 

	Quality-of-life improvement	Life extension
Technology P	0.2 × 8 = 1.6	10 years
Technology Q	0.5 × 8 = 4	8 years

These vectors can be used to make a kind of nuanced effectiveness analysis. Rather than establishing how much value one gets in one dimension from a certain cost, the vectors allow one to establish how much value one gets in two dimensions from a certain cost. Of course, this is merely an illustration. Quality-of-life improvement can be broken up into several dimensions, and other dimensions can be added.

Once one describes benefits with vectors as opposed to with unique numbers, a question arises concerning how to treat the vector. On the one hand, one might attempt to come up with some way of combining the different dimensions into an overall assessment that allows for rankings of all options. One can, e.g., add up values from dimensions and compare options with respect to the sum of values from different dimensions (for an approach similar to this, see [4]). On the other hand, one can move directly to the question of choice and ask what one should do given the situation as represented in the vector. The first approach allows one to construe more nuanced metrices of the value of health but cannot account for underlying incommensurability since it still provides weak orderings of all options. The second approach raises choice-theoretical questions.

Rather than conventional optimization, some other choice criterion must (sometimes) be used to actually rank options with respect to choiceworthiness when incommensurability is allowed for and vectors represent the value of options. If value incommensurability is possible among the top alternatives, there will be instances where there is no option that is at least as good as all alternatives (perhaps technologies P and Q). Of course, this does not mean that all options in a choice set containing incommensurable options will be permissible. Some options will be covered in the sense that there exists an alternative that is unambiguously better. In the expanded example below, technology Q- seems unambiguously worse than technology Q, and should not be chosen if the costs are the same:

**Table:** 

	Quality-of-life improvement	Life extension
Technology P	0.2 × 8 = 1.6	10 years
Technology Q	0.5 × 8 = 4	8 years
Technology Q-	0.5 × 8 = 4	7 years

In light of this, it could be suggested that rather than optimizing, effectiveness analysis should inform maximizing behavior, showing decision makers how they can make decisions that are at least *not worse* than any alternative [20, 25].

In many contexts where effectiveness analysis is used in healthcare planning and policy, the purpose is not to directly optimize, but rather to make sure that decisions are effective enough, a kind of satisficing (the National Institute for Health and Care Excellence (NICE) in the United Kingdom is a famous example [10]. Instead of having a satisficing approach to what medicines to include in a healthcare plan (e.g., at least 1 QALY per $30,000), those who embrace multidimensional analyses of benefits could have more nuanced satisficing approach such that options can satisfy the criterion in different ways. For instance, “include medicines in the healthcare plan if they *either* extend life with 1 year or generate quality-of-life improvements amounting to 1.5 per $30,000”.

A challenge that arises for this approach is that using effectiveness analyses that allow for value incommensurability on the side of benefits (or costs) actualizes a risk of what might be called value leakage [16, 19]. The problem occurs when one looks at sequences or sets of individually justified choices. A single decision maker who uses an incomplete effectiveness analysis and considers covered options impermissible can form a sequence of individually justified choices that generates less value than some other, available sequences of choices. A group of decision makers who use the same effectiveness analysis that admits of value incommensurability can form a set of individually justified choices that generates less value than some other, available set of choices. Although each individual choice is justified, the sequence or set of choices seems not justified.

Here is a very simple illustration of how this can happen:

*D*_*1*_ *D*_*2*_

*x*, *y x+*, *y*.

*D*_*1*_ and *D*_*2*_ are two choice situations, *x*, *y* and *x*+, *y* are the options in the respective choice situations. *y* is incommensurable in value with both *x* and *x+*, while *x +* is better than *x*. It is, in this constellation, permitted to choose *x* in *D*_*1*_ and *y* in *D*_*2*_, and thereby choosing a set of items {*x*, *y*} which is worse than an available set of items {*x+*, *y*}. If the approach is used in many choice situations, the problem can become substantial. Imagine that the only difference between *x* and *x* + is that *x* + is $10 cheaper than *x*. Choosing {*x*, *y*} rather than {*x+*, *y*} one thousand times means that one has lost $10,000, and more importantly, missed out on all the benefits one could have attained with that money.

In a sense, one might think of value leakage as a co-ordination problem. If the decisionmakers in *D*_*1*_ and *D*_*2*_ know that they are part of a whole that is in fact making two decisions, they would in effect be in a choice situation where they can choose between {*x*, *y*}, {*x+*, *y*} and {*y*, *y*}. In such a situation, they would not choose {*x*, *y*}. The issue is that the decisionmakers in *D*_*1*_ and *D*_*2*_ are unable to co-ordinate their behavior across the choice situations.

Another potential problem with using effectiveness analyses that allow for value incommensurability relates to how such approaches in more cases will group several options as top contenders. Rather than identifying a unique option that is more cost-effective than the alternatives, in many instances several options will be maximal in the sense that they are not worse than any available alternative. When decision makers have multiple options that are justified by the decision criteria, other things than the decision criteria will influence what option is chosen. This means, on the one hand, that discrimination and prejudice might become a bigger problem if one relies on effectiveness analyses that allow for value incommensurability [18]. On the other hand, it might remind policymakers of the importance of non-discrimination, sound judgment and good political processes.

## Preserving the structural features of conventional benefit-cost analysis

Although some items might be incommensurable in value, that does not mean that it is impossible to make comparisons of the items. Value incommensurability obtains when none of the conventional comparative relations determinately holds between two items *with respect to the relevant evaluative standard*, e.g., “objective value of health” or “community C’s valuation of health” [7]. Also when there is value incommensurability with respect to the relevant evaluative standard, one can ascribe a conventional comparative relation as holding between two items using some other evaluative standard. One can, for instance, establish that the works of Mozart are better than the works of Michelangelo with respect to expressing musical creativity. In fact, for all items that are incommensurable in value, it is true that a conventional comparative relation can be established if one changes the evaluative standard (at the extreme, one can compare all items with respect to evaluative standards like “how many words are needed to describe them”).

Invoking an entirely new evaluative standard to replace a standard that admits of value incommensurability does not make much sense. However, one can try to use an evaluative standard that respects what the initial standard establishes. In this vein, one could argue that although value incommensurability obtains between some items, those engaging in effectiveness analysis should act as if it does not and rely on conventional valuations of costs and benefits that make use of evaluative standards that rank all items. These valuations cannot be true to the actual value of costs and benefits (because sometimes there is value incommensurability), but they can be designed so that they are true to the actual comparative relations of costs and benefits when this is possible. They can preserve the conventional comparative relations that do obtain.

This idea can be compared to a well-known approach to vagueness. According to so-called supervaluationism, sentences that contain vague terms are “supertrue” if they are true on all *admissible precisifications* and “superfalse” if they are false on all admissible precisifications [11, 14]. An admissible precisification is a specification of the vague term that is in accordance with ordinary langue and respects so-called penumbral truths and connections. If we assume that Alan is definitely balder than Bob, but that it is indeterminate if Charles is balder than Alan and also indeterminate if Charles is balder than Bob (this is possible if Alan and Bob have similar patterns of hair distributions, while Charles is bald in a very different way), it is supertrue that Alan is balder than Bob and superfalse that Bob is balder than Alan. There are several admissible precisifications of “balder than”, but any precisification that entails that Bob is balder than Alan is inadmissible, since it fails to respect the penumbral connection that consist in Alan being balder than Bob.

If an evaluative standard fails to establish conventional comparative relations between all items so that some items are incommensurable in value, it might be suggested that decision- and policymakers ought to rely on some admissible precisification of the evaluative standard. For instance, if “the value of health” fails to rank all health states, decision- and policymakers ought to rely on a specification of “the value of health” that respects the penumbral connections that consists in conventional comparative facts that can be determined by “the value of health”, e.g., paraplegia is worse than a headache, the longer the duration of a health problem, the worse it is. There are many ways to specify a conception of “the value of health” that admits of value incommensurability, but some of them will be inadmissible.

There are some obvious practical upshots with this approach. First, it enables using very familiar decision-theoretical tools. Conventional expected utility theory requires valuations of goods and bads that rank all goods and bads. Similarly, conventional CBA and CEA can be used even if there is some value incommensurability among relevant items if one treats value incommensurability like this. By using admissible precisifications of evaluative standards, one avoids having to revise decision-theoretical tools.

Secondly, it is an approach that avoids problems such as value leakage. In the illustration of value leakage in the previous section, this approach would simply entail that choosing *x* in *D*_*1*_ and *y* in *D*_*2*_ is impermissible. Either *y* in *D*_*2*_ would be impermissible because *x* + is better than *y* according to the precisified evaluative standard, or *x* in *D*_*1*_ would be impermissible because *y* is better than *x* according to the precified evaluative standard. There is no admissible precisification of the evaluative standard that establishes that *y* is incommensurable in value with both *x* and *x +* while *x +* is better than *x* that says that *x* is at least as good as *y*, although *x +* is not at least as good as *y*. Such a specification of the standard would fail to respect penumbral connections and thereby be inadmissible.

However, the approach also actualizes questions. If value incommensurability really is present with respect to the evaluative standards that ought to be applied, relying on admissible precisifications of those evaluative standards means that one uses valuations of costs and benefits that fail to reflect the actual values that should be ascribed to different costs and benefits. This seems problematic for several reasons. First, if two items really are incommensurable in value, then there is no reason to favor one rather than the other, no reason to choose one rather than the other. By applying an admissible precisification of the evaluative standard that established the incommensurability, one will change this (except when the precisification establishes an equally as good as relation). This change in what one has reason to favor will appear for merely technical reasons, and thereby have very questionable justification. Instead of holding that there is no reason to favor *x* instances of depression over *y* instances of paraplegia (and vice versa), a commitment to decision-theoretical simplicity leads one to think *x* instances of depression is better than *y* instances of paraplegia (or vice versa). This seems unjustified.

Second, when value incommensurability is present, there will be several, mutually conflicting, admissible precisifications of the evaluative standard that respect penumbral connections. If we know that A is better than B, but that both A and B are incommensurable with C, there are two conflicting specifications of the evaluative standard that respect the penumbral connections. On one admissible precisification C is better than A, which is better than B. On a different admissible precisification A is better than B, which is better than C. Why, one wonders, should we use one of these precisifications rather than the other? If we are only interested in respecting penumbral connections, this question has no answer. It is entirely arbitrary which one we go for.

## Discussion

I have attempted to show that there are good reasons to accept that in many contexts in which effectiveness analysis is used, we have good reasons to accept that some benefits (or costs) are incommensurable in value in the sense that neither is more valuable than the other, nor are they equally as valuable. This would undermine conventional types of effectiveness analyses that measure benefits and costs with one-dimensional measurements that represent values with rational numbers (e.g., $ equivalents or QALY). The problem is straightforward: benefits and costs that are incommensurable in value cannot be weakly ordered, and rational numbers generate weak orderings of whatever it is they represent.

The paper discussed the two most obvious ways of dealing with this problem, neither of which is very appealing. First, one can revise the way one thinks about effectiveness analysis: abandon conventional approaches that represent the value of benefits and costs in unique dimensions and instead develop approaches that represent values with vectors. Such approaches can reflect underlying value incommensurability but actualize decision-theoretical problems. They are hard to use with standard decision theory and risk leading to value leakage, i.e., using them can lead to loss of value since they can justify forming sequences of choices that generate less value than other available sequences of choices.

Second, one can accept that there is underlying value incommensurability but hold on to conventional effectiveness analyses and continue to represent values with rational numbers. This approach by necessity misrepresents the value of benefits (or costs) as well as how these relate to each other. This is to accept that the method one uses *by necessity* is inaccurate and will generate inaccurate results. In practice, many (if not all) effectiveness analyses will contain inaccuracies stemming from methodological and epistemic difficulties figuring out how valuable different items are. A significant difference between such inaccuracies and the inaccuracies that are baked into conventional effectiveness analysis if value incommensurability obtains is that the former are contingent and can be improved upon whereas the latter are necessary and cannot be improved upon. For an approach that holds on to conventional effectiveness analyses even though value incommensurability is recognized, questions arise concerning why certain necessary inaccuracies are accepted rather than others, and also concerning how these decisions come about.

To some extent, the problem with value incommensurability for effectiveness analysis can be seen as just another issue that can be added to the long list of reasons to think of these analyses as very imperfect. It is well-known that one-dimensional measurements of benefits that are used in practice (e.g., QALY) are problematic. Insofar as they are built on surveys of people’s preferences, their reliability is undermined by problems with such surveys. How questions are framed affect respondents, selection of participants in surveys matter, and so on. Nevertheless, I believe one should not underestimate the uniqueness of the problem with value incommensurability. Whereas methodological problems are (in principle) solvable and incremental improvements can be made, value-theoretical problems such as value incommensurability cannot be addressed in the same way: an approach that is committed to weakly ordering all items cannot ever be tweaked so that it respects that some items cannot be weakly ordered. If methodological problems show that there in practice will be problems with conventional effectiveness analysis in the sense that it relies on mistaken valuations of benefits (or costs), value incommensurability implies that such mistaken valuations are parts and parcel of the effectiveness analysis.

## Data Availability

No datasets were generated or analysed during the current study.

## Footnotes

1 In addition to these types of differences between values, there are other value conflicts in the healthcare sector that run even deeper. For instance, sometimes decision makers will need to choose between what they think an unconscious patient wants and what they think is best for the patient. However, I am not aware of anyone who has even attempted to use effectiveness analysis in order to deal with these kinds of value conflicts.
